# Ecological Momentary Assessment and Intervention Principles for the Study of Awake Bruxism Behaviors, Part 1: General Principles and Preliminary Data on Healthy Young Italian Adults

**DOI:** 10.3389/fneur.2019.00169

**Published:** 2019-03-01

**Authors:** Alessandra Zani, Frank Lobbezoo, Alessandro Bracci, Jari Ahlberg, Daniele Manfredini

**Affiliations:** ^1^School of Dentistry, University of Padova, Padova, Italy; ^2^Department of Oral Kinesiology, Academic Centre for Dentistry Amsterdam (ACTA), University of Amsterdam, Vrije Universiteit Amsterdam, Amsterdam, Netherlands; ^3^Department of Oral and Maxillofacial Diseases, University of Helsinki, Helsinki, Finland; ^4^School of Dentistry, University of Siena, Siena, Italy

**Keywords:** awake bruxism, ecological momentary assessment, ecological momentary intervention, bruxism, smartphone, awake bruxism behaviors

## Abstract

**Background:** Awake bruxism (AB) is an oral condition that has some uncertainties concerning the epidemiology, also due to the different diagnostic strategies that have been adopted to address it in the research setting. The recent new definition of AB suggests that an ecological momentary assessment (EMA), which enables real-time reporting of the condition under study, can implement knowledge on the topic.

**Objectives:** This article will discuss the general principles of EMA and EMI (Ecological Momentary Intervention) and comment on a preliminary dataset gathered with a smartphone application in a population of Italian young adults.

**Materials and Methods:** A dedicated smartphone application has been used (BruxApp®) on a sample of 30 University students (mean age 24 ± 3.5 years) to record real time report on five specific oral conditions (relaxed jaw muscles, tooth contact, teeth clenching, teeth grinding, mandible bracing) that are related with the spectrum of AB activities. Data were recorded over a 7-day period for two times, with a 1-month interval between the two observation periods. The purpose of collecting data over a second week, 1-month later, was to monitor AB behaviors over time, and test for potential “EMI” effects.

**Results:** Over the first 7 days (T1), the average frequency of relaxed jaw muscles reports at the population level was 62%. Teeth contact (20%) and mandible bracing (14%) were the most frequent AB behaviors. No significant gender differences were detected. One month later, during the second week of data collection (T2), the frequency of the conditions was as follows: relaxed jaw muscles 74%, teeth contact 11% and mandible bracing 13%.

**Conclusions:** These data recorded do not allow any generalization due the unrepresentativeness of the study population. On the other hand, they can be used as templates for future comparisons to get deeper into the study of natural fluctuations of AB behaviors as well as into the potential biofeedback effect of an ecological momentary assessment/intervention. It is important to recognize that the use of smartphone technology may help to set range of values for AB frequency in otherwise healthy individuals, in order to stand as comparisons for selected populations with risk or associated factors.

## Introduction

Bruxism is an oral condition that is attracting interest from researchers and clinicians of several medical disciplines, such as neurologists, psychologists, dentists, physicians, and orofacial pain experts. The constantly evolving knowledge and the diverging approaches by the different specialties is reflected in the number of different bruxism definitions that has been provided over the past decades (1, 2).

In March 2017, a panel of invited experts took part to an International Consensus Meeting that provided separate definitions for sleep bruxism (SB) and awake bruxism (AB) and revisited the diagnostic grading that was previously proposed in 2013 by the same panel. The introduction of a specific definition for AB is of particular interest, especially considering the paucity of epidemiological data. AB has been defined as “a masticatory muscle activity during wakefulness that is characterized by repetitive or sustained tooth contact and/or by bracing or thrusting of the mandible and is not a movement disorder in otherwise healthy individuals” (3).

Such definition has implications concerning the diagnosis, especially in the light of the influence that the adoption of different diagnostic strategies may have on the prevalence data reported for both adults (4) and children/adolescents (5). In short, the consensus paper suggests that bruxism should be considered as a jaw-muscle behavior that must be measured in its continuum to achieve a definite diagnosis. As such, the muscle behavior should be measured at best with continuous electromyographic (EMG) recordings during wakefulness (6). On the other hand, it should be recognized that acquiring hour-long EMG recordings while awake is difficult for obvious technical reasons as well as the discomfort for the patients. For this reason, an alternative option to AB assessment, viz., the Ecological Momentary Assessment (EMA), has been suggested.

EMA refers to real time report of a behavior, a feeling, or whichever condition under study (7). It was developed in the 1980 s as a way of addressing the limitation of traditional quantitative methods in psychological science (8). In short, over a time frame within the course of his/her daily affairs, an individual is prompted at fixed or random time points to answer questions about what he/she is currently doing and/or experiencing. Doing so, multiple recording points during the day, close in time to the experience in the natural environment, are allowed (9). The fact that data are collected in the everyday (“real world”) environment, as subjects go about their lives, increases their representativeness and this ecological aspect allows generalization to an individual's real life. Several studies on the natural course of AB could be performed using EMA strategies as an instrument to investigate the day-to-day variability of behaviors over multiple observation periods (e.g., during stressful periods that may have an influence on the individuals' current psychological wellbeing).

Based on the definition of AB provided in the consensus paper, using EMA in the bruxism field will help achieving a better description of AB epidemiology, both at the general population level and in selected group of individuals with purported risk factors for additive AB and with possible clinical consequences. Within these premises, this two-part article will discuss the general principles of EMA and comment on a preliminary dataset gathered in Italian young adults with a dedicated smartphone application (part 1), and will describe the translational efforts into Polish language as part of an ongoing multicenter research on AB epidemiology (part 2).

## Current application of EMA principles

EMA strategies are being used to study the prevalence of a wide spectrum of conditions in behavioral medicine [e.g., smoking cessation, alcohol consumption, eating disorders (7)]. In addition, they are useful to conduct specific studies on daily habits to monitor a behavior over time, such as in the case of a mobile app supported by the American Sleep Apnea Association to study the correlation between sleep habits and some illnesses (e.g., diabetes, heart pathologies, obesity, depression) (10). As far as oral behaviors are concerned, EMA has already proven reliable in the research setting (11), but it should be remarked that EMA-based data on AB are fragmental and limited to a few investigations on selected behaviors, such as tooth clenching and tooth contact habits (9, 12). The simplest EMA strategy is the daily diary, which is still commonly used in several clinical medical fields and has also been used to gather the few data available on AB so far. However, with the widespread development of mobile electronic technologies, new ways to approach EMA in the research and clinical settings have become available. Interestingly, the use of technology has introduced a new possible way for clinicians to engage patients from a therapeutic viewpoint as well [i.e., Ecological Momentary Intervention (EMI)] (8). In other words, the progressive adaptation of EMA techniques to the technological progress has *de facto* opened a new era for EMA (9) and created more therapeutic opportunities (i.e., EMI).

Over the past few years, the most commonly used devices were palmtop computers and mobile phones (7), but today there is no doubt that smartphones offer the best potential in terms of customized applications and internet access. Smartphones diffusion is widespread in all age groups, so they are useful to capture real-time experiences in potentially huge study samples. EMA protocols based on smartphones typically require participants to complete assessments at predetermined time intervals or windows, either in response to specific states or events or in response to an auditory signal programmed by the researchers. Thus, smartphones provide an ideal platform for real-time reports at multiple daily recording points over multiple-day spans.

There are only few studies on the application of EMA principles in the dental field. In the field of orofacial pain, the only available findings suggest that non-functional tooth contact (i.e., tooth contact during activities that are not associated with normal function, such as reading books, watching television, working, etc.), recorded at 20-min intervals for 10 days, are more prevalent in patients with temporomandibular disorders (TMDs) than in healthy subjects (12).

With the purpose to get deeper into the knowledge on awake bruxism, an application (form here on abbreviated as “app”) based on EMA principles was recently developed (BruxApp®, BruxApp Team, Pontedera, Italy). The app sends alert sounds at random times during the day. The smartphone user must answer in real time by tapping on the display icon that refers to the current condition of the jaw muscles or teeth position: relaxed jaw muscles, tooth contact, tooth clenching, tooth grinding or jaw clenching [without tooth contact (i.e., mandible bracing)]. Such behaviors were selected as they that are part of the AB spectrum. For further details on the software, readers are referred to the original publication (13, 14).

## Preliminary smartphone-based EMA report on AB and data discussion

A preliminary study on AB using BruxApp has been performed in a population of undergraduate University students, on a sample of 30 otherwise healthy young adults (9 males, 21 females; mean age 24 ± 3.5 years) during the period from October 2015 to March 2016. All subjects were undergraduate students attending different University courses (e.g., school of Dentistry, Medicine and Surgery, Law, and school of Engineering). Data were recorded over a 7-day period for two times, with a one-month interval between the two observation periods. Data were collected by the leading author of this manuscript (A.Z.) and stored in an excel database. The study was approved by the Institutional Review Board of the University of Padova, Italy, and all participants signed a written consent to take part to the study.

This set of preliminary data is an example of two potentially separate investigations. During the first week, smartphone-based EMA has been used to get an overview of the frequency of each oral condition (i.e., relaxed jaw muscles, mandible bracing, teeth clenching, tooth contact, teeth grinding). On the other hand, it has been theorized that being asked about a behavior in close contextual and temporal proximity to its occurrence draws one individual's attention toward the behavior, thereby promoting self-awareness and potentially inducing positive changes with respect to the capability to self-recognize and avoid the behavior (i.e., EMI-biofeedback) (15). Thus, the purpose of collecting data over a second week, one-month later, was twofold, viz., (1) to monitor AB behaviors over time, and (2) to test for potential “EMI” effects. The app was programed to send 15 alerts per day during the 8.00–22.00 h span, with individually set breaks during lunchtimes. In order to reduce the possibility that individuals may modify their behavior based on the expectation of receiving the alert at predetermined time intervals or hours, alerts were randomly generated by the app. Over the first seven days, the average frequency of relaxed jaw muscles reports at the study population level was 62%. Teeth contact (20%) and mandible bracing (14%) were the most frequent AB behaviors. No significant gender differences were detected. One month later, during the second week of data collection, the frequency of the conditions was as follows: relaxed jaw muscles 74%, teeth contact 11% and mandible bracing 13%. (Table 1)

**Table 1:** Mean values of frequency data of positive observations (standard deviations in parenthesis) expressed in percentage for the different AB behaviors over the 7-day observation period (T1) and the second period of observation after a month (T2).

**Activity**	**T1**	**T2**
Relaxed	62 (34.9)%	74 (30.0)%
Teeth clenching	3 (9.5)%	2 (5.8)%
Teeth contact	20 (22.5)%	11 (19.5)%
Teeth grinding	1 (0.4)%	1 (0.6)%
Mandible bracing	14 (20.1)%	13 (26.0)%

*Data refer to a study population-level average*.

Data collected during the second week suggested an increase in the frequency of relaxed jaw muscles' condition and a decrease in teeth contact habit, thus indicating a certain degree of variability of AB behaviors over time on one hand, but also potentially supporting an EMI effectiveness on the other hand. These preliminary ideas and dataset recorded in a small, selected population of university students do not allow any generalization. Nonetheless, they can be used as templates for future comparisons to get deeper into the study of natural fluctuations of AB behaviors as well as into the potential biofeedback effect of an ecological momentary assessment approach to patients with clinically relevant bruxism.

## Discussion

Until now, most available data on AB prevalence have been obtained by means of retrospective self-reporting at a single observation point (4). Such approach requires the participants to recall to their minds the frequency of a habit over the timespan covered by the report (e.g., days, weeks, months, and years) and give a generic answer. The resulting answers can be biased due to reporting errors (16, 17). Such shortcoming can easily be overcome with EMA, which requires participants to report close on time with the current experience. Moreover, EMA occurs in natural settings, thus offering a potential advantage in terms of generalizability and ecological validity of findings with respect to hour-long EMG recordings during wakefulness.

The above-outlined general framework can be refined and the EMA usefulness can be maximized by using smartphones apps, which are so diffused that they generally do not even require an explanation on how to use them thanks to a user-friendly interface (18, 19). Smartphone apps provide a pre-programmed or randomly generated auditory signal that recalls the patient's attention to the behavior under investigation several times per day. An additional advantage is that apps allow tracking compliance rate. All in all, this means that the outcome variable can be assessed multiple times and researchers are enabled to collect huge amounts of information on the epidemiological features, risk factors, and clinical consequences of the condition under study (6, 20).

As for awake bruxism, a basic premise to understand the potential usefulness of smartphone-based EMA is that the 2018 definition highlights two important aspects. First, focus has been definitively shifted to the muscle activity, specifying that bruxism does not necessarily involve tooth contact (21). Second, it must be remarked that in “otherwise healthy people,” bruxism is not a pathological condition. From an etiological viewpoint, bruxism can be a sign of a disorder [e.g., obstructive sleep apnea, psychological or neurological disorders (22)]. From a clinical perspective, it may be a risk factor for negative consequences (e.g., tooth wear, muscle fatigue and pain, failures of dental restorations) or even a factor associated with positive health outcomes [e.g., reducing the risk of detrimental chemical tooth wear by increasing salivation in patients with gastro-esophageal reflux (23)].

The above considerations suggests the need to re-conceptualize bruxism as a spectrum of behaviors, which need to be more extensively studied from an epidemiological perspective than in the past. As for AB, the basic requirement is to gather information on the frequency of all conditions of the behavioral spectrum (i.e., tooth contact habits, tooth clenching, tooth grinding, mandible bracing) and to possibly identify a range of “normal values” for AB in healthy individuals in a natural environment. The second step should be to collect data in selected groups of subjects with associated conditions and risk factors for additive AB (e.g., stress sensitivity, anxiety).

Currently, knowledge on AB is very limited as far as prevalence or frequency data on healthy individuals are concerned. In particular, there is an almost complete lack of information on mandible bracing. Within this framework, the development of a smartphone app to definitively introduce EMA into the field of AB research (EMA/AB) is a much required strategy to implement knowledge on the epidemiological features of AB by studying the natural course and fluctuations of signs, symptoms, and exposure to etiological factors.

An app-based EMA approach is also suitable for collecting consistent data across different investigations and within multiple observation points of the same study, thus making it a promising option to perform longitudinal and multicenter researches. The ultimate goal is to approximate the “definite” assessment of AB in the clinical setting.

Using a smartphone app (i.e., BruxApp) in a population of healthy young adults has allowed to gather preliminary data on the average frequency for the different AB behaviors (i.e., tooth contact, mandible bracing, tooth clenching, tooth grinding) over two 7-day observation periods. Tooth contact habits and jaw clenching were the most frequently reported conditions, with an average frequency for the “relaxed jaw muscles” answer of 62% at the study population level. One month later, data gathered over a second observation week showed a 74% frequency, potentially showing both natural fluctuation and a self-awareness effect. The paucity and inconsistency of the available literature on this topic makes it impossible to compare these findings with previous studies.

In an effort to standardize all the investigations performed with BruxApp, a Research Version has thus been developed. It has a pre-set number of alerts programmed per day (viz., 20) as well as the possibility to access the raw application data, without any filters to automatically discard “low-compliance” days. Thus, it can be easily hypothesized that these findings, which show the potential of smartphone-based EMA in the research setting, will soon be part of a wide international dataset on the frequency of AB.

In the clinical setting, the app has a potential usefulness beyond the scope of gathering epidemiological data for research purposes. Indeed, an individual's awareness, behavior, and experience are altered when he/she are knowingly being assessed (13). Smartphone-based EMA can minimize this potential bias, since it allows the patient to focus his/her attention to the focal behavior only at random moments and in the natural environment, thus avoiding any laboratory setting biases. Nonetheless, focusing repeatedly on a specific behavior might enable the patient to improve self-awareness, which is responsible in promoting more control and cognitive change (24) (i.e., biofeedback/EMI). Thus, the app may have a rationale for use as a potential therapeutic strategy in myofascial pain patients with a self-reported history of awake bruxism.

The term EMI provides a framework for treatments characterized by the delivery of interventions when people go about their daily lives (25, 26). In other words, the treatment setting is the real world. Smartphone-based apps open the possibility to perform EMI treatments in many forms, ranging from basic clinical recommendation and counseling to more formalized and structured interventions. (27) EMI benefits of extending the treatment beyond the standard context, providing support in patients' daily routine. Thus, the potential for the use of smartphone/EMA approach as a strategy to implement cognitive-behavioral management of AB is worth an assessment within the framework of multimodal conservative treatment (28).

Such an app-based cognitive-behavioral approach is also interesting as for the amount of information on awake bruxism and its possible consequences that can be conveyed to the patients via the smartphone display. This could implement an individual's understanding of the need to keep the jaw muscles relaxed. Thus, there are enough elements to hypothesize that an app that instructs patients on the various consequences of bruxism, with an alert biofeedback function such as sound pulses at random times that remind patients about keeping their jaw muscles relaxed, may have interesting clinical potential. Support to this suggestion comes from encouraging literature findings on other conditions. Indeed, EMI based on the biofeedback mechanism has already been used effectively in people who show potentially dangerous behaviors as a strategy to recognize and modify it. Within the last several years, interventions have been delivered to patients with a variety of conditions and health behaviors, such as eating and anxiety disorders (10), smoking habits (7, 29, 30), and psychological distress (31), and have been even used to facilitate prevention behavior amongst HIV-infected individuals (32, 33).

As for the indications, it must be recognized that, at the moment, there are no specific recommendations on when and how to prescribe the use of EMA method in clinical practice. On the other hand, it can be suggested that a twofold strategy of prescription should be refined: (1) to assess the frequency of AB behavior (i.e., EMA), and (2) to implement the control of AB in patients with potential clinical consequences (i.e., EMI). The two prescriptions are consequential, based on the logical diagnosis-to-treatment pathway, and can be equally adopted in both the research setting and the clinical setting. On the other hand, research findings gathered over the next few years will be fundamental to design treatment-need algorithms based on the frequency of AB behaviors in patients with clinical signs and symptoms. Amongst those, myofascial pain patients with stress sensitivity and anxiety personality are the theoretical best-fitting targets of app-based EMI due to the potential influence on emotion-related mandible bracing.

Considering the above, BruxApp has been translated into several languages to ease consistency of cross-cultural investigations, and it is currently used in an international multicenter research project on the epidemiology of awake bruxism. The project involves more than 20 countries worldwide, and its description is one of the topics of the second part of this manuscript.

## Conclusions

This article discussed the possible application of ecological momentary assessment and intervention principles to the study of awake bruxism behaviors by adopting smartphone technology. Among the possible EMA strategies, the development of smartphone-based applications seems a promising strategy to get deeper into the evaluation of several epidemiological, etiological, and management issues concerning AB.

Future EMA researches on the frequency of AB activities (e.g., teeth clenching, mandible bracing, teeth grinding, teeth contact) in healthy individuals and on their additive frequency in selected populations with comorbid conditions (e.g., psychological and social impairment, orofacial pain, sleep disorders) will shed light onto these behaviors and will contribute to a better understanding of this complex topic.

## Author Contributions

All authors listed have made a substantial, direct and intellectual contribution to the work, and approved it for publication.

### Conflict of Interest Statement

The authors declare that the research was conducted in the absence of any commercial or financial relationships that could be construed as a potential conflict of interest.
